# Building a “Hello World” for self-driving labs: The Closed-loop Spectroscopy Lab Light-mixing demo

**DOI:** 10.1016/j.xpro.2023.102329

**Published:** 2023-05-31

**Authors:** Sterling G. Baird, Taylor D. Sparks

**Affiliations:** 1 Materials Science & Engineering Department, University of Utah, Salt Lake City, UT 84108, USA

**Keywords:** High Throughput Screening, Chemistry, Material sciences, Computer sciences

## Abstract

Learn how to build a Closed-loop Spectroscopy Lab: Light-mixing demo (CLSLab:Light) to perform color matching via RGB LEDs and a light sensor for under 100 USD and less than an hour of setup. Our tutorial covers ordering parts, verifying prerequisites, software setup, sensor mounting, testing, and an optimization algorithm comparison tutorial. We use secure IoT-style communication via MQTT, MicroPython firmware on a pre-soldered Pico W microcontroller, and the self-driving-lab-demo Python package. A video tutorial is available at https://youtu.be/D54yfxRSY6s.

For complete details on the use and execution of this protocol, please refer to Baird et al.^1^

## Highlights

• Learn self-driving laboratories and lab automation with a simple demo
• Use in hands-on classroom settings and as an example for public outreach
• Use as a prototyping tool for research applications and self-driving lab concepts
• Accompanied by a video build tutorial on YouTube

## Abstract (editor highlights)

Publisher’s note: Undertaking any experimental protocol requires adherence to local institutional guidelines for laboratory safety and ethics.

## Graphical abstract

**Figure:**
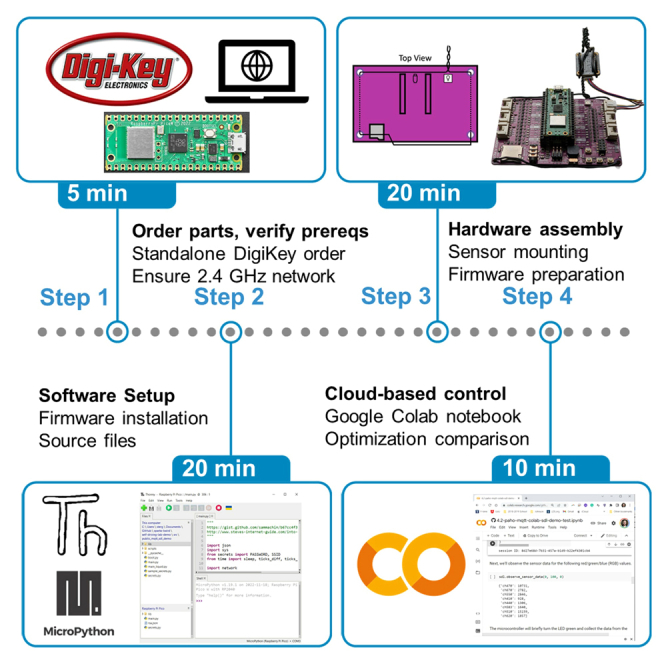

## Before you begin

The protocol below describes how to set up Closed-loop Spectroscopy Lab: Light-mixing Demo (CLSLab:Light), a “Hello, World!” for a “self-driving” (i.e., autonomous) laboratory (SDL)^2^ using a Pico W microcontroller, LEDs, a light sensor, and Bayesian optimization. CLSLab:Light incorporates key principles for SDLs including sending commands, receiving sensor data, physics-based simulation, and advanced optimization. This “Hello, World!” introduction is accessible to students, educators, hobbyists, and researchers for less than 100 USD, a small footprint, and under an hour of setup time. For a full video build tutorial, please refer to https://youtu.be/D54yfxRSY6s. There are some deviations between the instructions in the YouTube video build tutorial and recent versions of the self-driving-lab-demo Python package. In particular, see steps 13 and 14.

### Order required parts

**Timing: 5 min (not including shipping time)**

**Figure 1:**
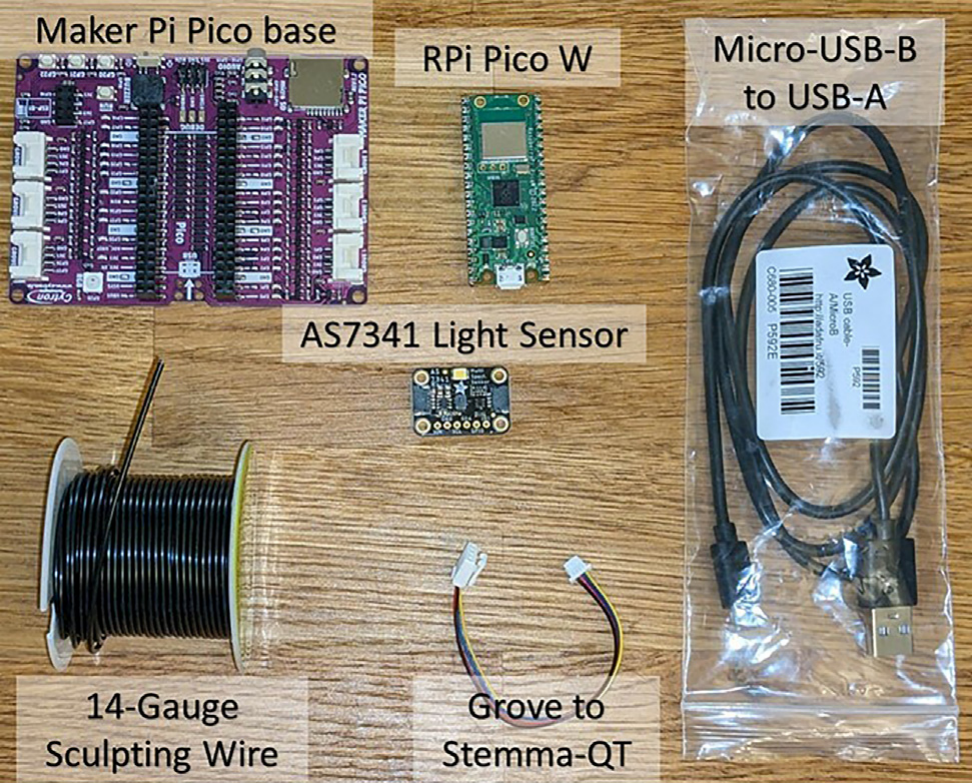
Visual bill of materials

***Note:*** For the first option, the total is 68.61 USD (or 73.72 USD including optional parts) + shipping as of 2022-03-06.

***Note:*** The authors plan to periodically check and update the “DigiKey Order" link at https://hackaday.io/project/186289-autonomous-research-laboratories in case of part shortages or deprecation.

***Note:*** In case of part shortages, many products may also be found on the Adafruit website.

1. Order the parts: (https://www.digikey.com/short/qztj2jt7 AND Pico W with pre-soldered headers) OR https://www.digikey.com/short/vtzjbvr2. A visual summary of parts is given in Figure 1.

**CRITICAL:** If you’d like to avoid soldering, you will need to source a Pico W with headers or a Pico WH separately, such as PiShop’s Pico W’s with pre-soldered headers. See also Raspberry Pi's supported resellers for the Pico W.

***Note:*** The sculpting wire needs to be 14 gauge (2 mm) or thinner, including the insulation jacket, and rigid enough to support the sensor. The sculpting wire is only used for mounting purposes, not to conduct electricity. Sculpting wire is also available at Amazon. Approximately 3′ is required. See problem 5.

***Note:*** The purpose of the wall adapter is so that, after initial setup, the demo can be powered standalone where communication happens purely via Wi-Fi.

***Note:*** The hardware and software was designed to work with the Pico W, though the setup can be adapted for other microcontrollers. See problem 1.

***Note:*** The bill of materials, not including the sculpting wire, is also available at Adafruit.

### Additional prerequisites

**Timing: N/A**

**CRITICAL:** Ensure access to a 2.4 GHz Wi-Fi network (SSID + password).

***Note:*** The purpose of using a wireless connection rather than a hardwired one is to capture the principles behind “cloud experimentation”, where the host and the client may be separated by large geographical distances. Additionally, this allows for a computer to only be required for initial setup such that the device can function standalone, waiting to receive commands and send sensor data. This captures best practices of a scaled-up cloud-accessible lab or network of labs. For more context, see https://github.com/sparks-baird/self-driving-lab-demo/discussions/91 and https://github.com/sparks-baird/self-driving-lab-demo/discussions/62. For links to a simple example using a wired connection and related discussion, see problem 2.

***Note:*** The Pico W only supports 2.4 GHz Wi-Fi networks. See self-driving-lab-demo #76 for additional context and recommendations on setting up a 2.4 GHz Wi-Fi network, if not already available.

***Note:*** WPA enterprise networks such as Eduroam and other networks that use captive portals (most schools, coffee shops, etc.) are not yet supported by MicroPython. It needs to be a network such that on a computer, you can click on the Wi-Fi name (SSID), enter the password, and click connect (no additional steps). Check to see if your institution offers network support for internet of things devices (e.g., ULink at University of Utah).

***Note:*** Home networks can have both a 5G and a 2.4 GHz network (e.g., “My Network 5G” and “My Network”).

**CRITICAL:** If you use a mobile hotspot, you may need to use your device’s “extended compatibility” feature to drop the mobile hotspot from 5G to 2.4 GHz. See also prepaid, long-expiry hotspot and classroom demos with standalone network access discussions, which includes a summary of recommendations for prepaid mobile hotspots.

2. Ensure access to a computer (for initial setup only).

***Note:*** At a minimum, the computer needs to be able to run the Thonny editor (lightweight) and it must have at least one USB-A port.

3. If the headers are not already soldered onto the microcontroller, ensure access to a soldering iron and soldering wire (thinner is better in this case).

**Optional**: Ensure the Pico W can successfully connect to a computer by holding the BOOTSEL button on the Pico W while connecting the Pico W to your computer via the USB cable. If a new drive appears, that indicates that the Pico W is working normally.

***Note:*** If soldering, be careful to only heat the gold pads to avoid damaging the circuitry.

## Key resources table

**Table:** 

REAGENT or RESOURCE	SOURCE	IDENTIFIER
**Deposited data**		

Red, Green, and Blue LED Spectral Data	Baird and Sparks^1^	https://github.com/sparks-baird/self-driving-lab-demo/tree/v0.8.2/src/self_driving_lab_demo/data

**Software and algorithms**		

self-driving-lab-demo v0.8.2	Baird and Sparks^1^	https://github.com/sparks-baird/self-driving-lab-demo

**Other**		

AS7341 Color Sensor	DigiKey (Adafruit Product)	Cat#1528-4698-ND
Grove to Stemma-QT adapter	DigiKey (Adafruit Product)	Cat#1528-4528-ND OR Cat#1528-4528-ND
Raspberry Pi Pico W with pre-soldered headers OR (Raspberry Pi Pico W AND Header pins with 20 positions and 2.54 mm pitch (x2))	PiShop OR (DigiKey-Adafruit Product AND DigiKey-Amphenol CS)	Cat#ASM-1918 OR (Cat#2648-SC0918CT-ND AND Cat#10129378-920001BLF-ND)
USB-A to USB-B Cable	DigiKey (Adafruit Product)	Cat#380-1431-ND
Maker Pi Pico base (without Pico)	DigiKey (Adafruit Product)	Cat#3614-MAKER-PI-PICO-NB-ND
AC/DC Wall Mount Adapter 5V 5W	DigiKey (Adafruit Product)	Cat#1470-2768-ND
18 AWG Hook-up solid black wire, 100’ (Outer diameter 14 AWG or higher)	DigiKey (Remington Industries)	Cat#2328-18UL1007SLDBLA-ND
Terminal Binding Post M2.5 (Optional)	DigiKey (Keystone Electronics)	Cat#36-8737-ND
128MB Micro SD Memory Card (Optional)	DigiKey (Adafruit Product)	Cat#1528-5250-ND

## Step-by-step method details

### Hardware setup

**Timing: 20 min**

Unless pre-soldered, attach the headers onto the Pico W, mount the light sensor so that the pinhole is facing the red green blue (RGB) LED, connect the light sensor to the board, and get the microcontroller ready for firmware installation.

1. Unless pre-soldered, Solder headers onto the Pico W or use a hammer header pin install rig for Pico W.

***Note:*** If soldering, insert the Pico W headers into the Maker Pi Pico base, place the Pico W on top of the headers, and solder the headers to the Pico W (MagPi guide, Tom's hardware guide, or YouTube video), and remove the Pico W from the Maker Pi Pico base.

***Note:*** Pico install rigs are not compatible with the Pico W. It must be labeled explicitly as “Pico W”.

**Figure 2:**
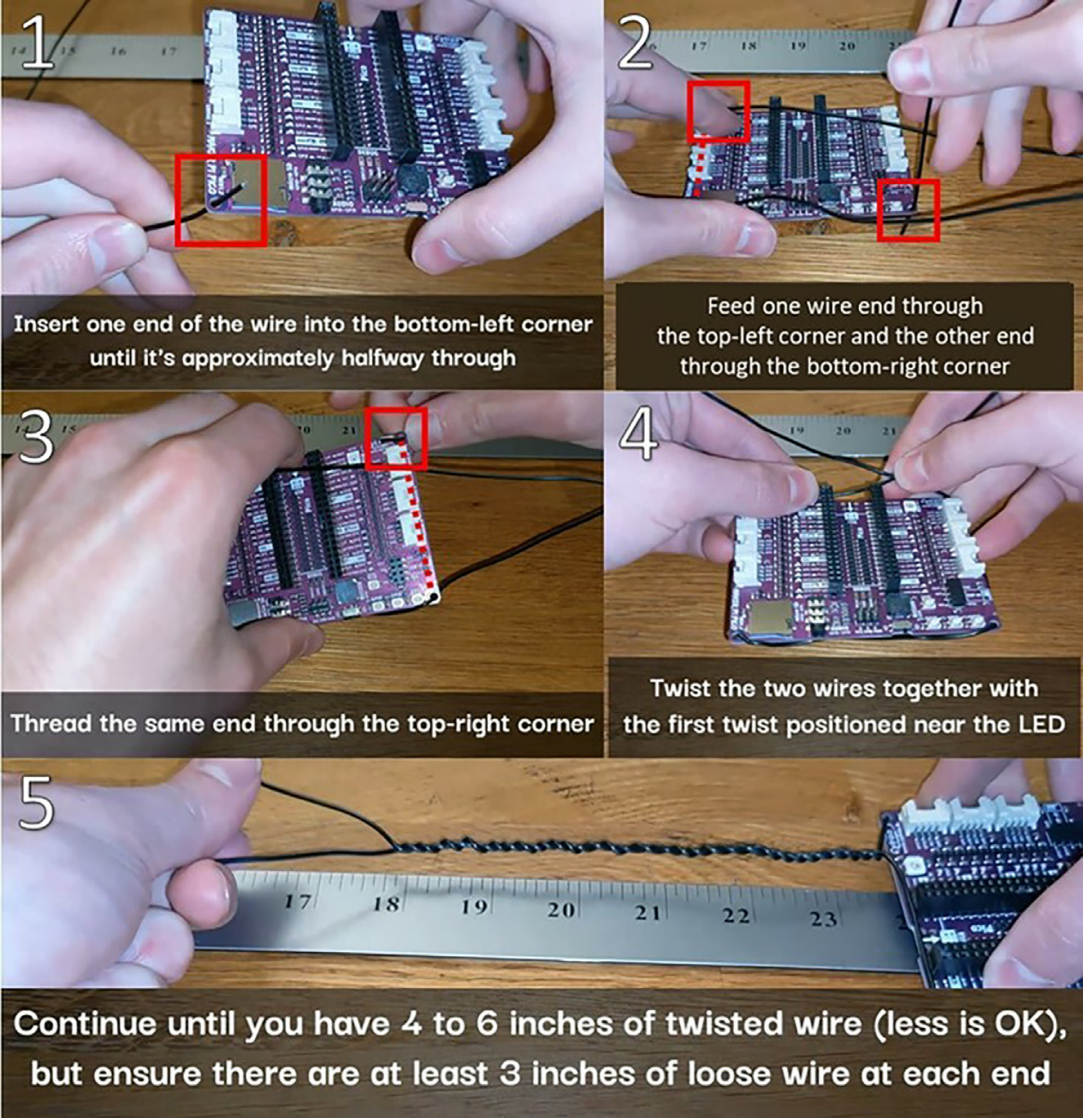
Wire mounting instructions

**Figure 3:**
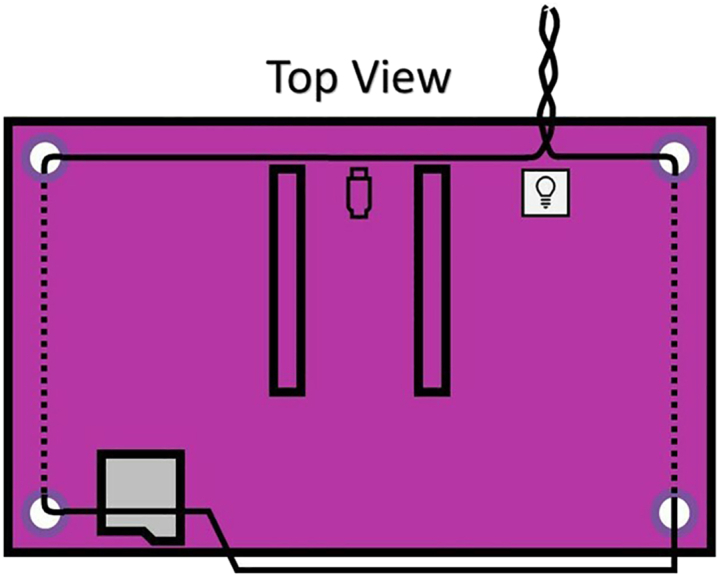
Wire mounting schematic

***Note:*** This setup will allow the position and orientation of the sensor to be both adjustable and steady.

2. Prepare 3 feet of sculpting wire (cut with wire cutters or bend until it breaks)
3. Thread the sculpting wire through each mounting hole on the Maker Pi Pico base, then twist the wires together near the RGB LED. See Figures 2 and 3, and Methods video S1.

4. Continue twisting until you have 4–6 inches of twisted wire, and ensure that there are at least 3 inches of loose, untwisted wire at each end. See Figure 2 and Methods video S1.

***Note:*** (the leftover, untwisted wire will be threaded through the mounting holes of the light sensor in the next step). For a more modular alternative of fixturing the wire ends to the Maker Pi Pico base, see problem 4.

5. Thread the same sculpting wire through the AS7341 light sensor and position the sensor so the pinhole is facing approximately 3–4 inches away from the RGB LED. See Figure 4 and Methods video S2.

**Figure 4:**
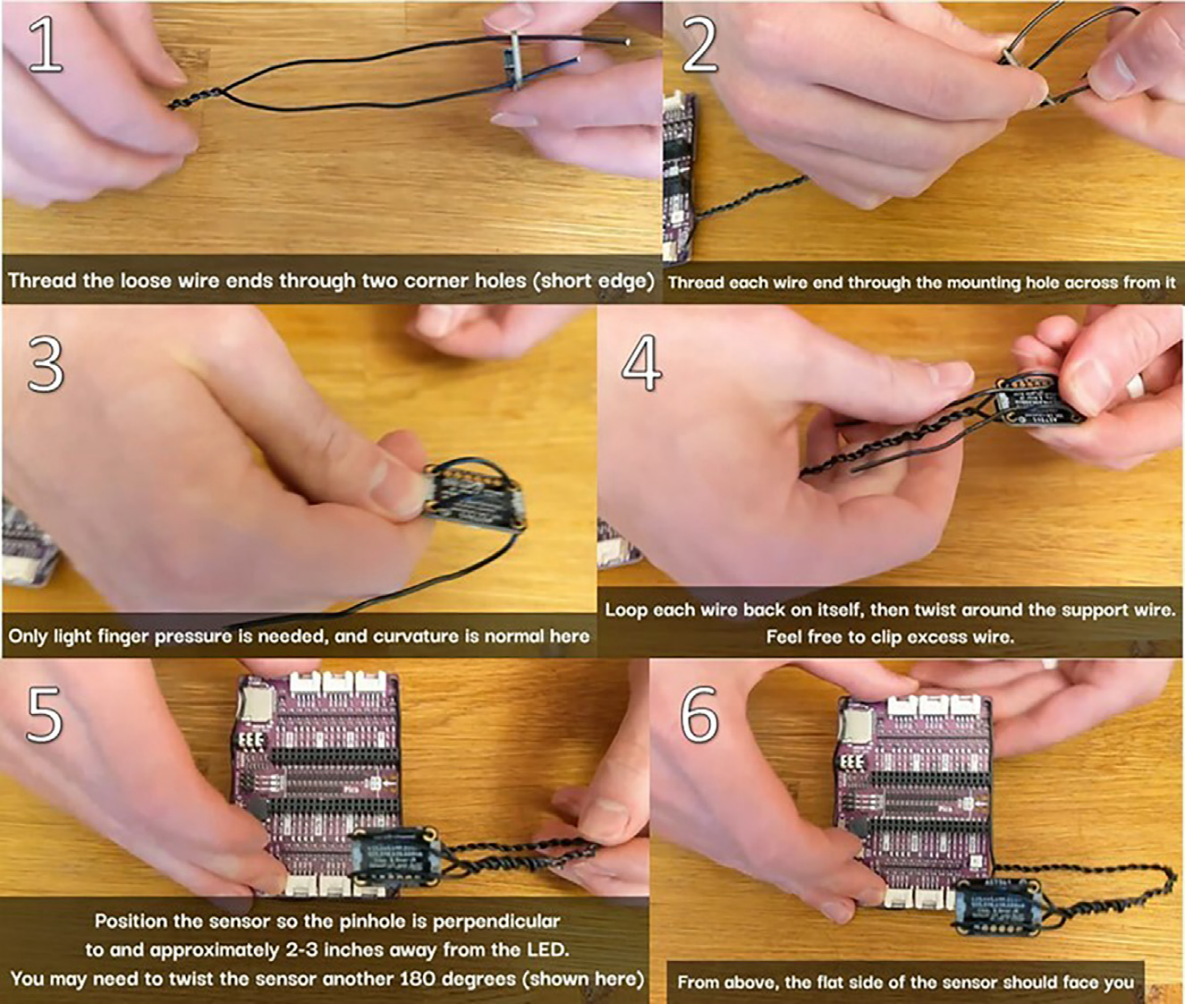
Light sensor mounting instructions
6. Connect the Grove/Stemma-QT connector into Grove port 6 (GP26&27) and the AS7341, insert the SD card (**optional**), insert the Pico W, and while holding the BOOTSEL button, connect the Pico W to the computer. See Figure 5 and Methods video S3.

**Figure 5:**
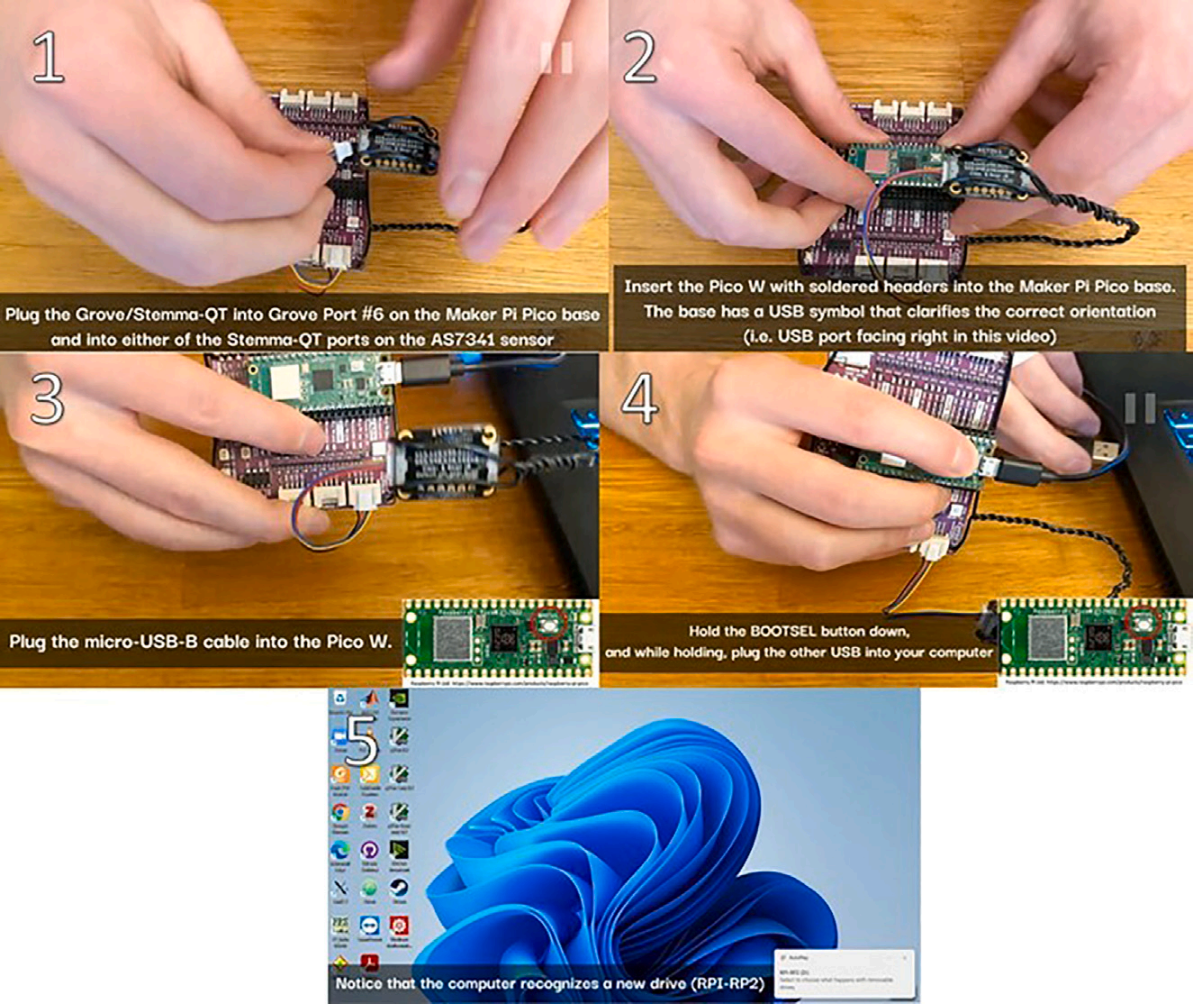
Hardware connections

Methods video S1. Thread the mounting wire through the mounting holes of the Maker Pi Pico base, see steps 3 and 4

Methods video S2. Thread the remaining mounting wire through the mounting holes of the AS7341 light sensor and position the sensor above the LEDs, see step 5

Methods video S3. Attach the Pico W and the AS7341 light sensor to the Maker Pi Pico base, then connect the USB cable from the Pico W to the computer while holding down the BOOTSEL button, see step 6

### Software setup

**Timing: 20 min**

Install the MicroPython firmware onto the Pico W microcontroller, enter the Wi-Fi credentials, and upload the source code files.

7. Download and install Thonny, a Python IDE with native support for microcontrollers, onto your computer. See Methods video S4.
  a. Choose the platform appropriate for you (in my case, this is Windows 64-bit, Python 3.10).
  b. When installing, use the default settings: "Standard (default)". Thonny comes with its own version of Python located by default at C:\Users\<username>\AppData\Local\Programs\Thonny\python.exe on Windows computers.
  c. It is not anticipated that this will cause conflicts with existing installations of Python; however, for conda users, an isolated installation may be performed via the following commands in a conda shell:

conda create -n sdl-demo-thonny python==3.10.∗

conda activate sdl-demo-thonny

pip install thonny

thonny

8. Click on the lower-right dropdown and click "Install MicroPython", which will install the microcontroller firmware onto the Pico W. See Figure 6 and Methods video S4.

**Figure 6:**
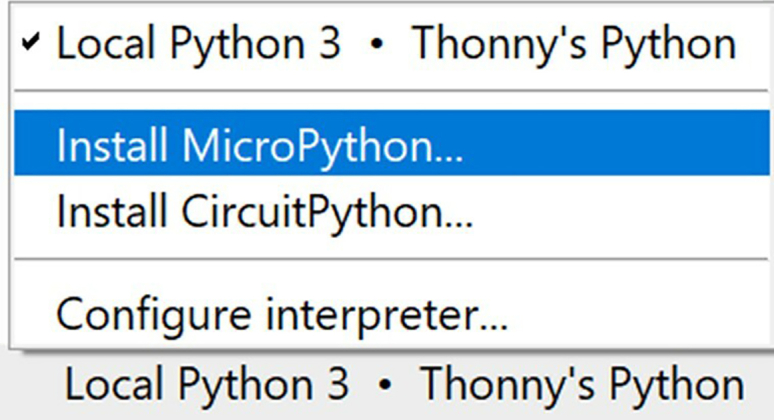
Firmware installation dropdown
9. Choose "MicroPython variant: Raspberry Pi - Pico W / Pico WH" and click install. See Figure 7 and Methods video S4.

**Figure 7:**
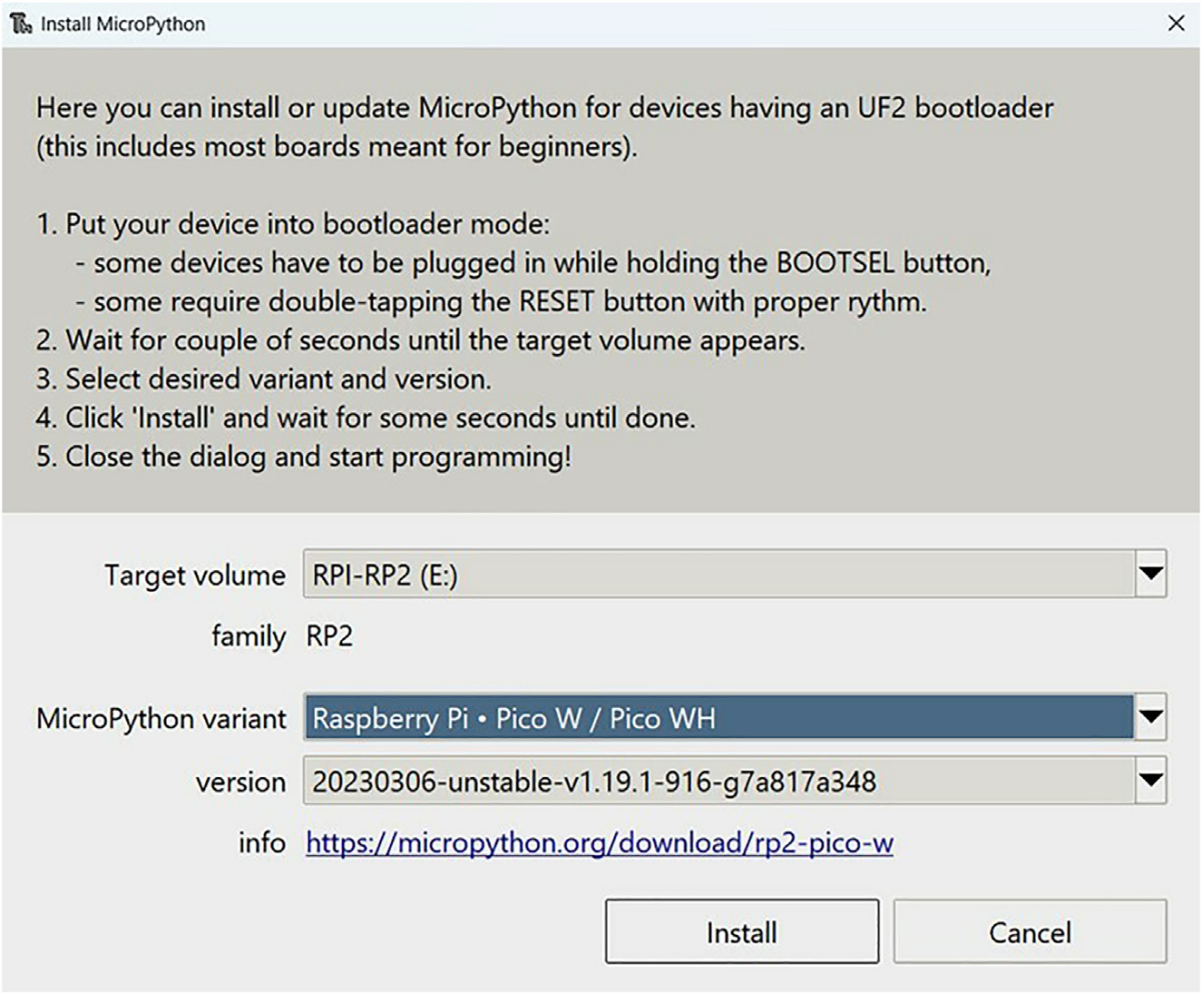
MicroPython installation dialogue box
10. Change the interpreter from Local Python 3 to MicroPython (Raspberry Pi Pico), which will open a shell that can be used to enter MicroPython commands that run directly on the Pico W. See Figure 8 and Methods video S4.

**Figure 8:**
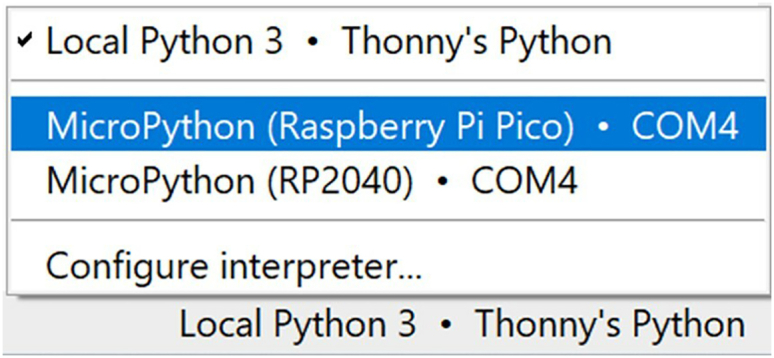
Interpreter dropdown
11. In Thonny’s menubar, click "View" then "Files" to open a sidebar which shows both your local computer’s files (top) and the files on the Pico W (bottom). See Figure 9 and Methods video S5.

**Figure 9:**
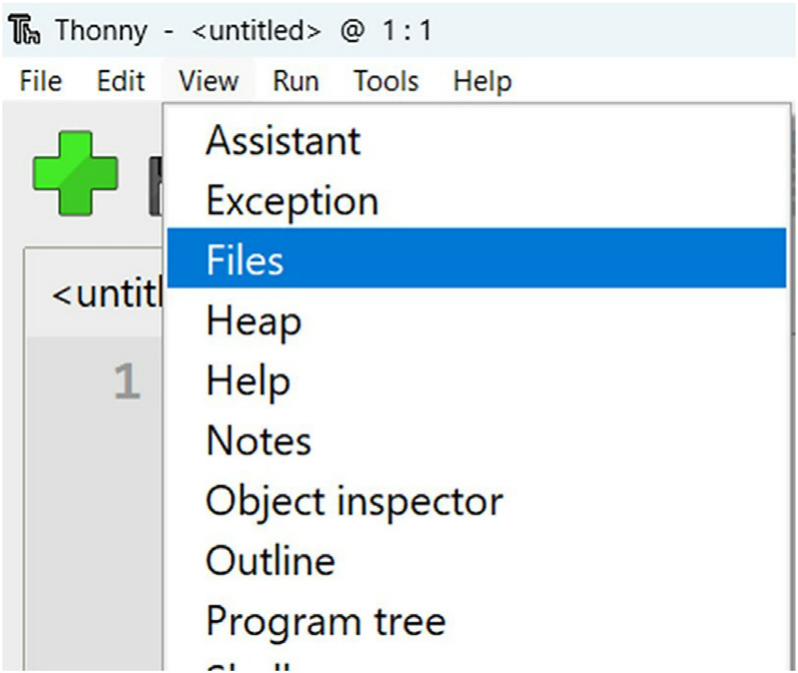
Opening the files sidebar
12. Download *sdl_demo.zip* from the latest release at self-driving-lab-demo to your computer and unzip it. See Methods video S5.
13. In Thonny, navigate to the unzipped *sdl_demo* folder, open *secrets.py*, enter your Wi-Fi network name (SSID) and password as Python strings, and save *secrets.py.* See Figures 10 and 11, and Methods video S5.
  ***Optional:*** you can create your own MongoDB Atlas database and enter values for MONGODB_API_KEY, MONGODB_COLLECTION_NAME, and DEVICE_NICKNAME into *secrets.py* (see below).
  ***Optional:*** you can create your own HiveMQ instance and enter *secrets.py* credentials for HIVEMQ_USERNAME, HIVEMQ_PASSWORD, and HIVEMQ_HOST (see below).
  a. Set up a MongoDB database backend.
    ***Note:*** If ignored, the demo will function, just without logging data to a database (i.e., the user becomes responsible for saving the data on the client side). See problem 3.
    i. Create an account at https://www.mongodb.com/cloud/atlas/register.
    ii. Create a free, Shared Cluster. See Figure 12.
      ***Note:*** optionally rename Cluster0 to something of your choice, e.g., self-driving-labs. You can leave the default provider as-is.

**Figure 12:**
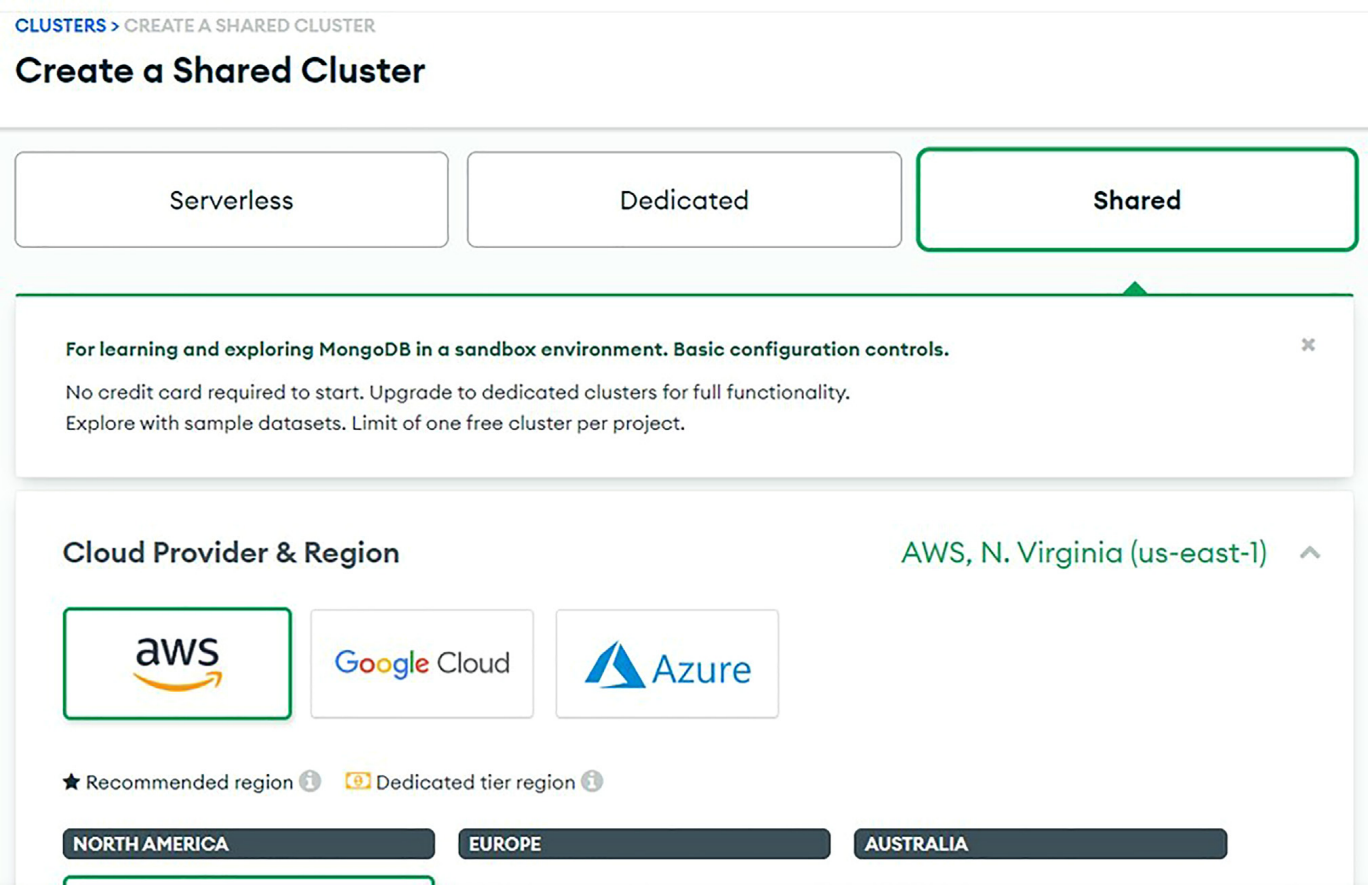
Setting up a MongoDB shared cluster
    iii. Navigate to “Data Services” → “Deployment” → “Database” and click “Browse Collections”. See Figure 13.

**Figure 13:**
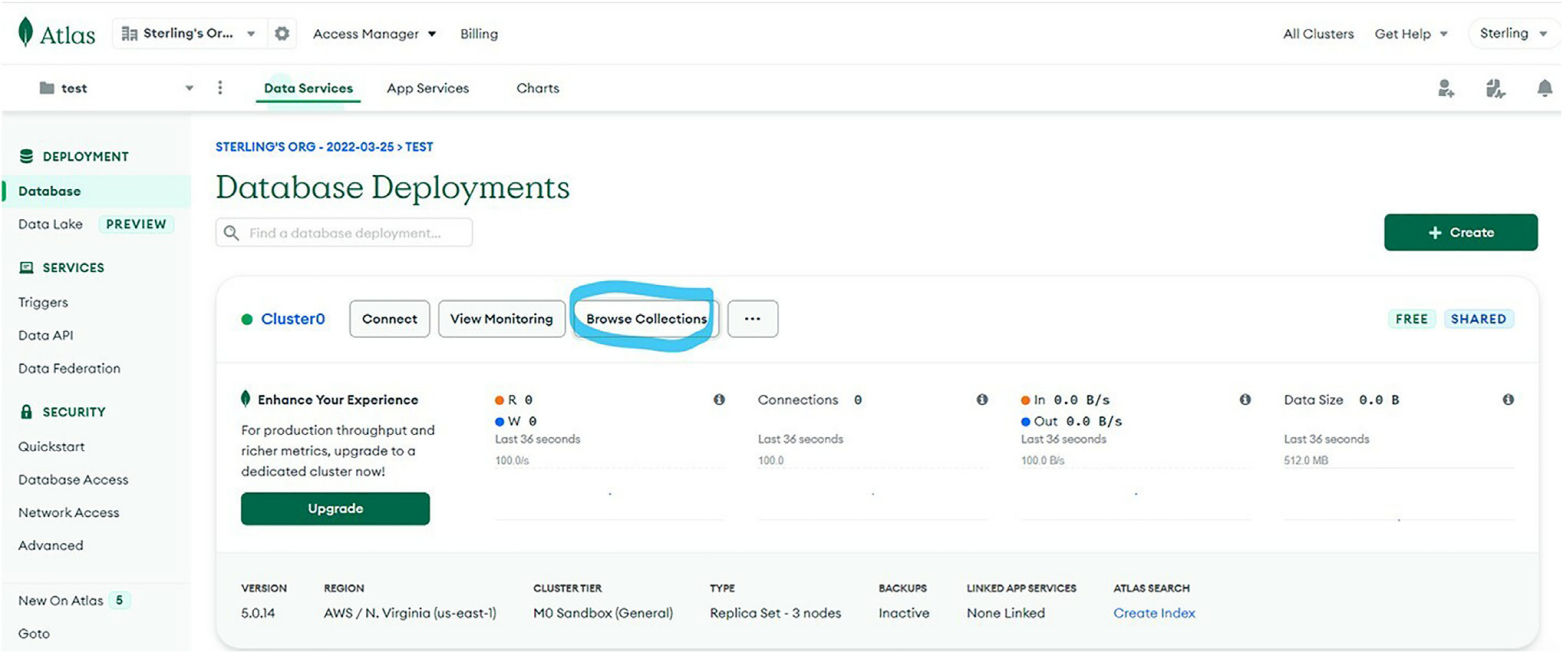
Create a MongoDB database
    iv. Click “Add My Own Data”
    v. Enter a database name (e.g., clslab-light-mixing) and collection name (e.g., test).
    vi. Copy the names into MONGODB_DATABASE_NAME and MONGODB_COLLECTION_NAME in secrets.py.
    vii. Navigate to “Data Services” → “Services” → “Data API”, use the dropdown to select your cluster, and click “Enable Data Access from the Data API”. See Figure 14.

**Figure 14:**
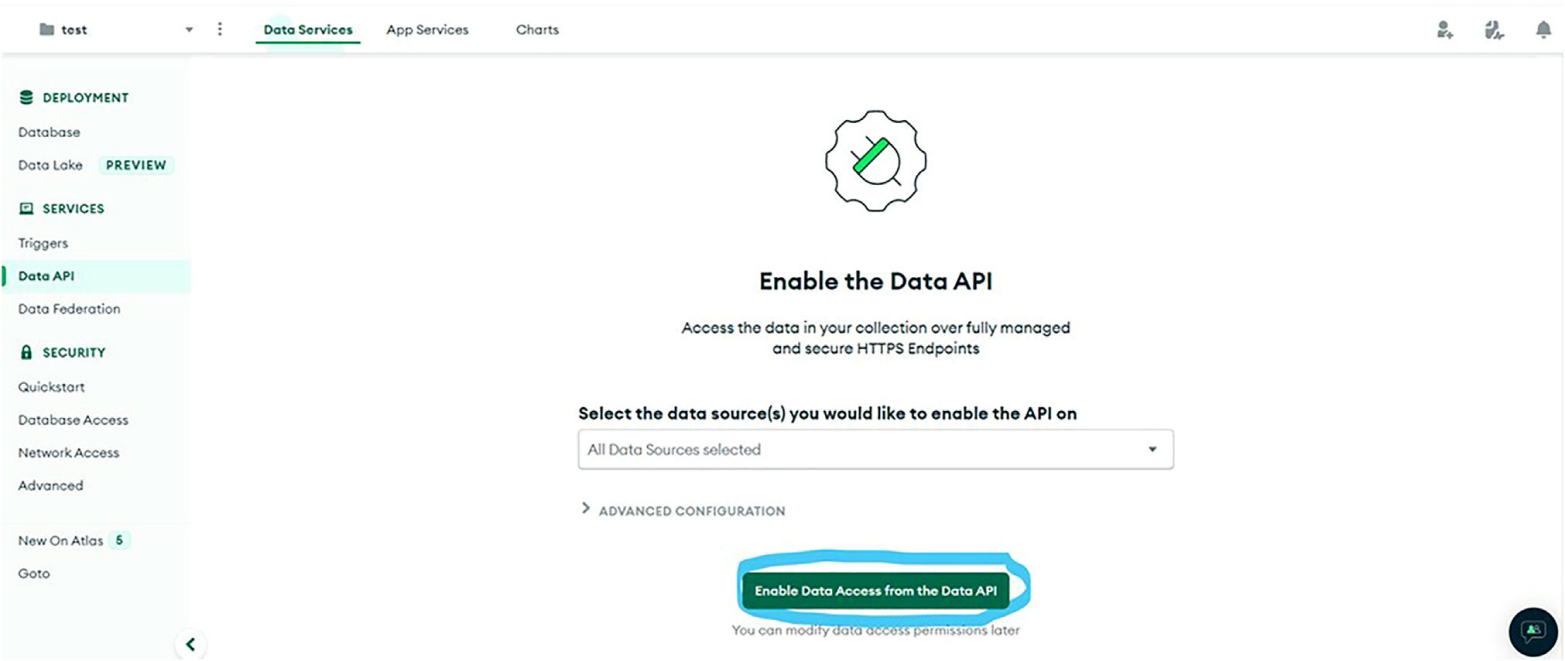
Enable the Data API
    viii. Note the app name in the “URL Endpoint” box of the form “https://data.mongodb-api.com/app/<data-abc123> /endpoint/data/v1” where <data-abc123> is the app name. See Figure 15.

**Figure 15:**
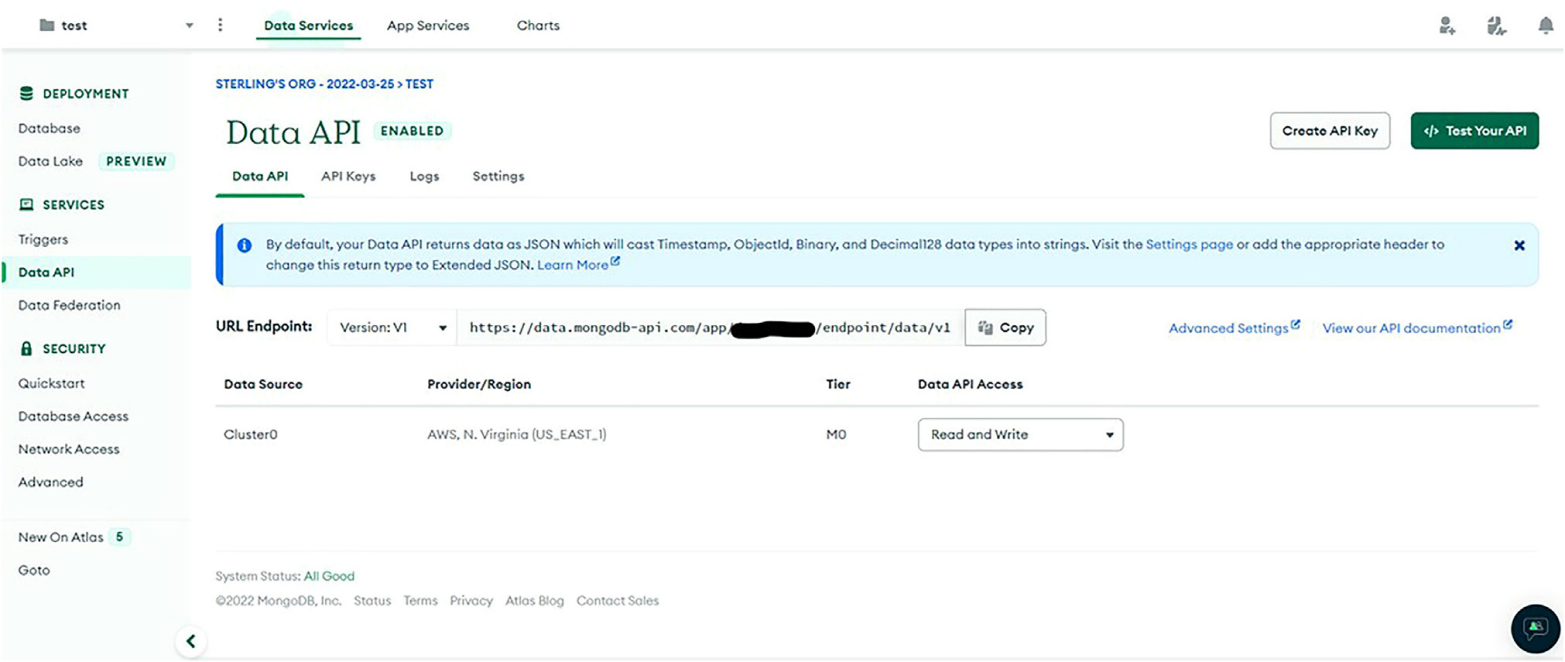
Retrieve MONGO
    ix. Copy the app name into the MONGODB_APP_NAME variable in secrets.py.
    x. Click “Create API Key”, enter a name of your choice (e.g., clslab-light), and click “Generate API key”. See Figure 16.

**Figure 16:**
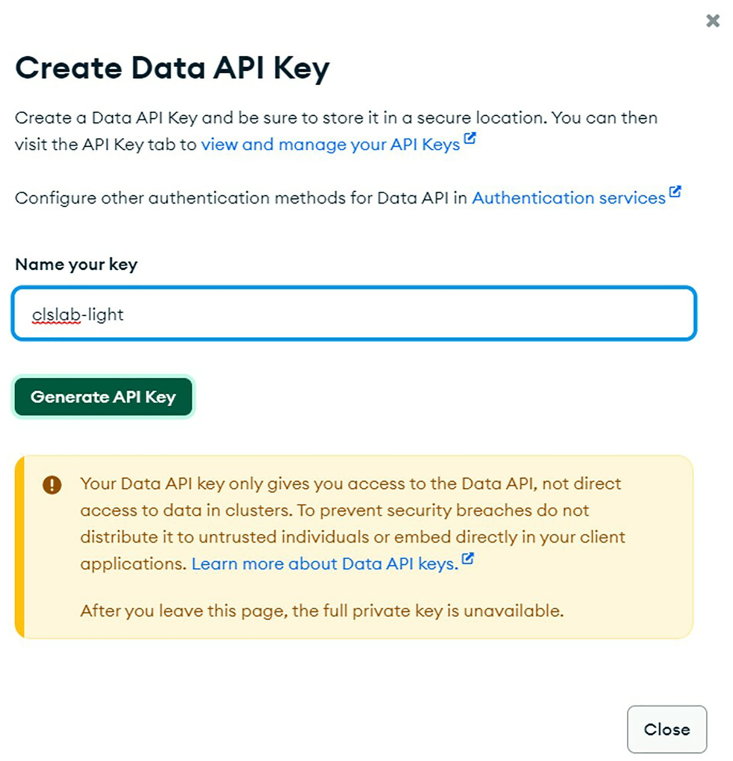
Create Data API key
    xi. Copy the API key and store it somewhere secure, then paste the API key into the MONGODB_API_KEY variable in *secrets.py.*
  b. Create your own HiveMQ instance.
    ***Note:*** If this setup is ignored, the demo will function properly; however, the hardware commands and sensor data will be transmitted via a default HiveMQ instance for which the credentials are public. Setting up your own HiveMQ instance ensures that the data you transfer remains private and secure. Other MQTT brokers such as Mosquitto or Adafruit IO are available. At the time of writing, we recommend HiveMQ because it provides free instances with generous limits. Setting up a private MQTT broker is in line with best practices for internet of things (IoT) security and should be used especially when working with sensitive data.
    i. Navigate to https://www.hivemq.com/mqtt-cloud-broker/, click “Try out for free”, and create an account.
    ii. Set up credentials by entering a username and password and press “ADD”. See Figure 17.

**Figure 17:**
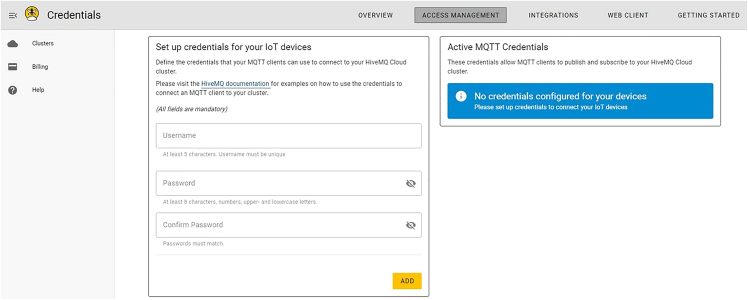
Set up HiveMQ credentials
    iii. Navigate to the “Clusters” tab and copy the URL (e.g., abc123.s2.eu.hivemq.cloud) to HIVEMQ_HOST in *secrets.py.* Also update HIVEMQ_USERNAME and HIVEMQ_PASSWORD with the username and password from the previous step. See Figure 18.

**Figure 18:**
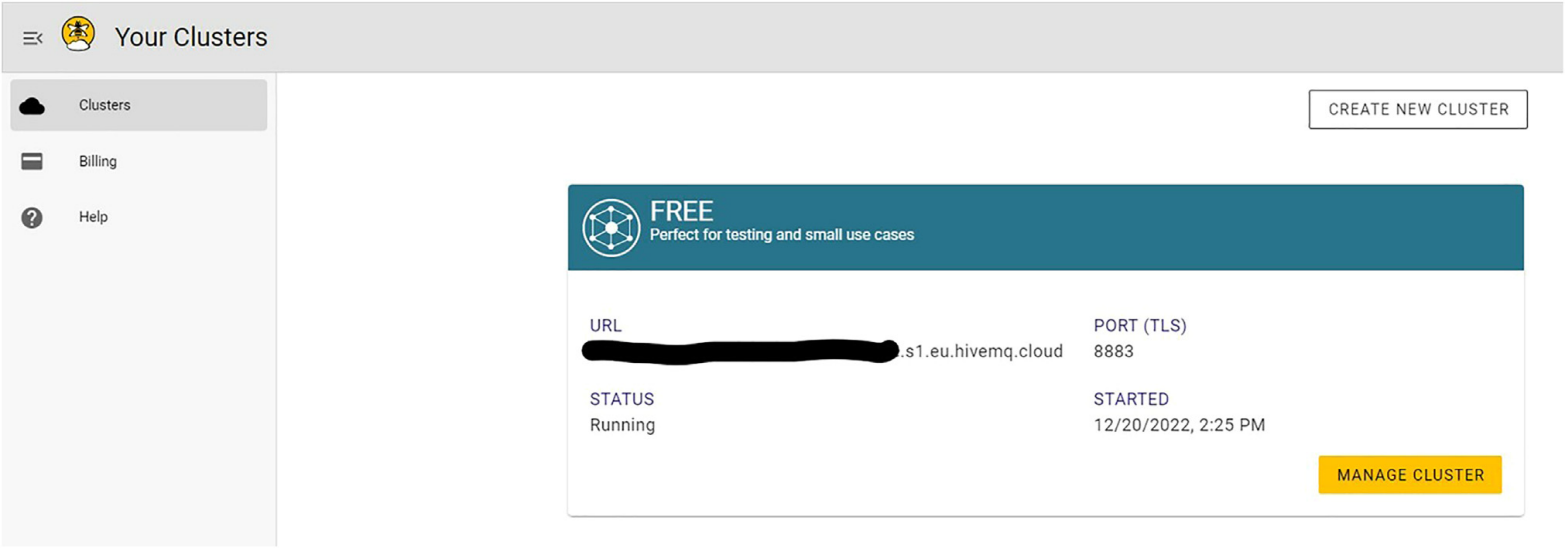
Locate the HiveMQ host URI
    iv. Create a certificate using the Google Colab notebook at https://github.com/sparks-baird/self-driving-lab-demo/blob/v0.7.3/notebooks/7.2.1-hivemq-openssl-certificate.ipynb.
      ***Note:*** This file is used to do secure authentication via HiveMQ.
    v. Enter the server address (i.e., HIVEMQ_HOST) into *secrets.py* and run the Google Colab cells.
    vi. Follow the instructions to download the hivemq-com-chain.der file to the unzipped sdl_demo folder.

**Figure 10:**
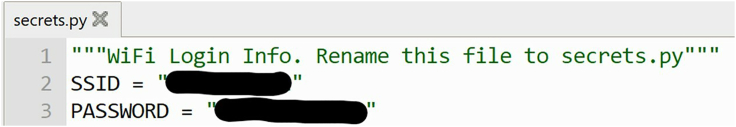
Editing secrets.py

**Figure 11:**
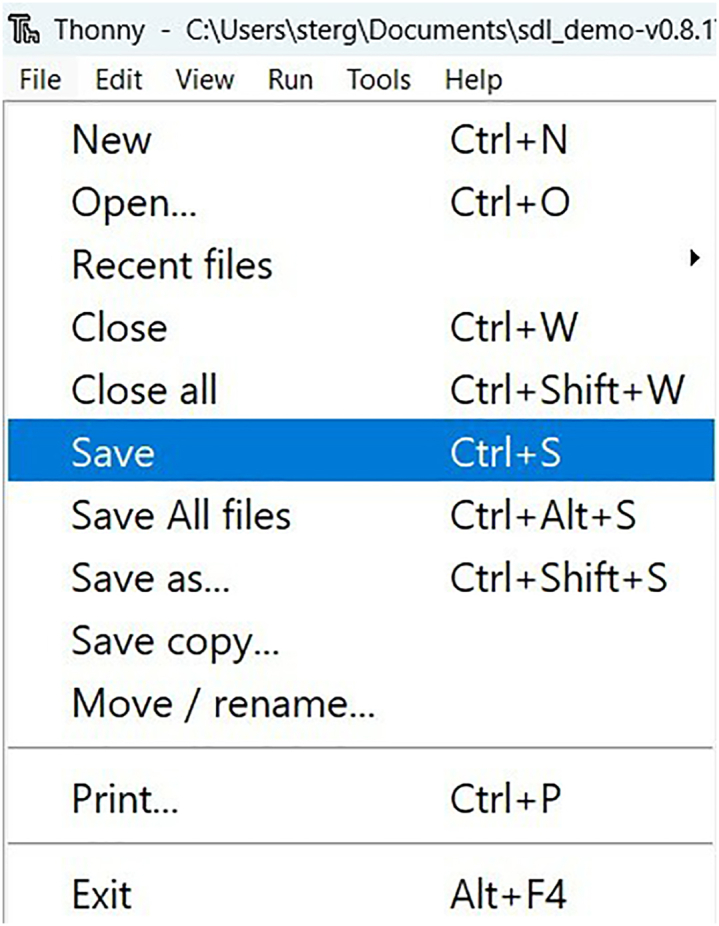
Saving secrets.py
14. Upload files to the Pico W microcontroller. See Figure 19 and Methods video S6.
  a. While holding Ctrl (Windows) or Cmd (Mac), select "lib", "main.py", “hivemq-com-chain.der”, and "secrets.py"
    ***Note:*** hivemq-com-chain.der is not mentioned in the YouTube tutorial, as it was not implemented at the time of creating the video.
  b. Right click in the gray region.
  c. Click "Upload to /".

**Figure 19:**
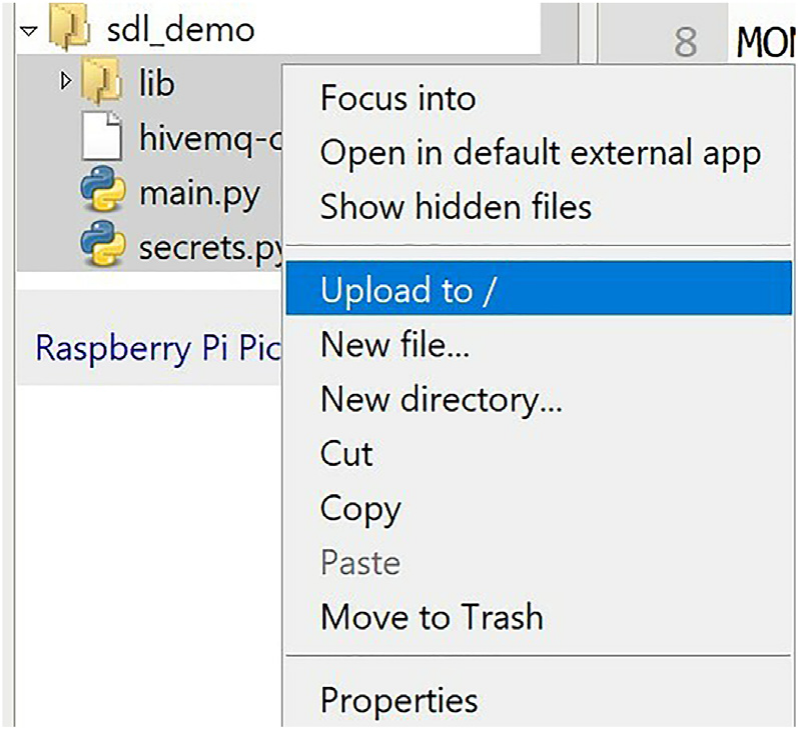
Uploading source files to microcontroller
15. Double click to open *main.py*, click the green play button (i.e., run the code on the Pico W), and note the PICO ID that prints to the command window ("prefix/picow/<PICO_ID>/"). See Figure 20 and See Methods video S6.

**Figure 20:**
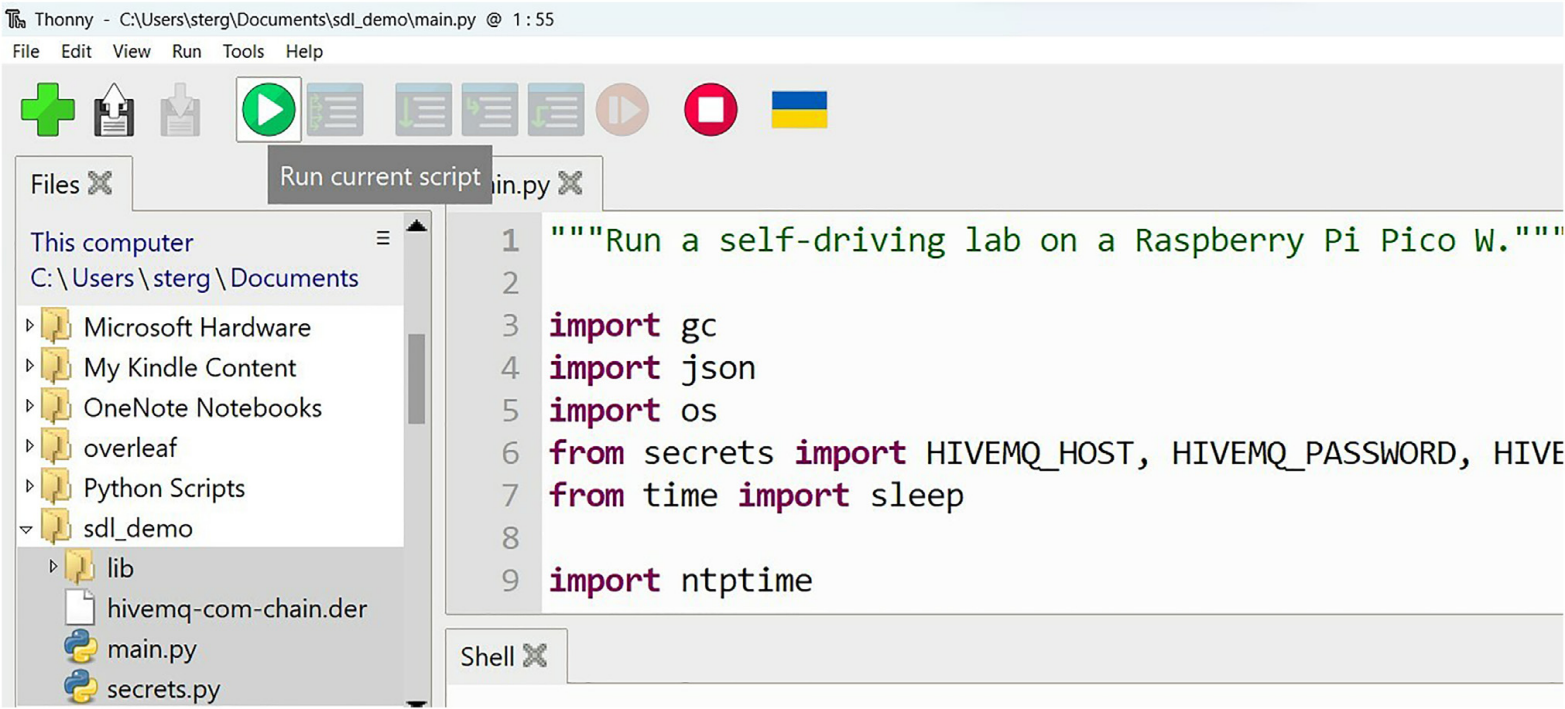
Running main.py

**CRITICAL:** main.py needs to be run on the Pico W microcontroller (host), not on your local machine (client).

***Note:*** This will act as the “password” to control the demo.

Methods video S4. Download the Thonny editor and install the MicroPython firmware onto the Pico W, see steps 7, 8, 9, and 10

Methods video S5. Download the source code from GitHub, unzip it, and enter Wi-Fi credentials, see steps 11, 12, and 13

Methods video S6. Upload the source code to the Pico W and run the main.py script, see steps 14 and 15

### Control from the cloud

**Timing: 10 min**

Control the device via internet-of-things style communication (MQTT) and run a basic optimization comparison of grid search vs. random search vs. Bayesian optimization.

16. Open notebooks/4.2-paho-mqtt-colab-sdl-demo-test.ipynb in Google Colab. See Methods video S7.
17. Scroll to the first code cell and click the play button to install the self-driving-lab-demo Python package. See Figure 21 and Methods video S7.

**Figure 21:**
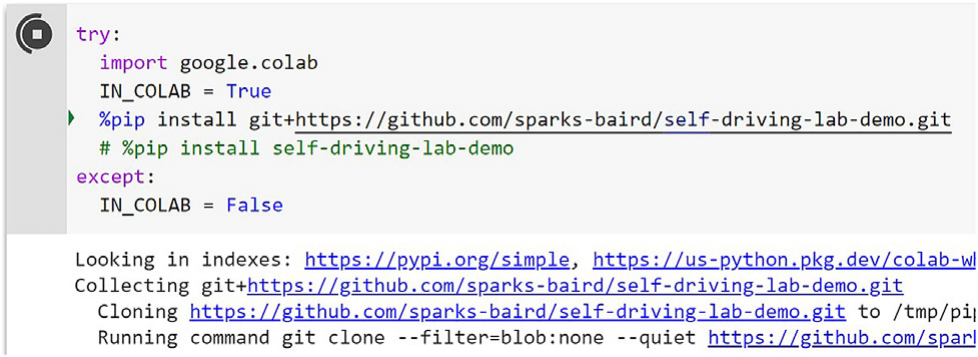
Python package installation. You can install the latest version from the main branch via `pip install git+https://github.com/sparks-baird/self-driving-lab-demo.git` or the most recent stable version via `pip install self-driving-lab-demo`.
18. Copy the PICO ID from the Thonny editor and paste it in place of "test" (without quotes). See Figure 22. An example image of the output is given in Figure 23. See also Methods video S8.

**Figure 22:**
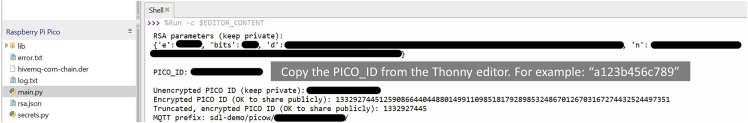
Copying the Pico ID from the Thonny editor

**Figure 23:**
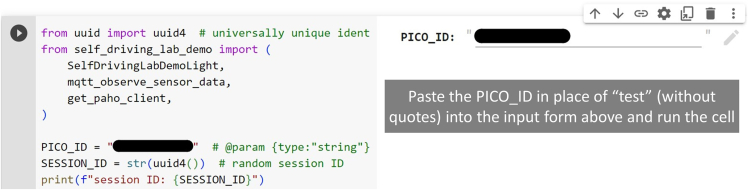
Pasting the Pico ID into the Google Colab form box

***Note:*** the actual output to the command window may vary in future releases.

***Note:*** If you leave PICO_ID set to “test”, this will control a public demo maintained by the authors for testing and demonstration purposes. The authors will strive to keep this public test demo available for the foreseeable future with minimal downtime.

19. Run the remaining code cells. See Methods video S8, S9, and S10.
  a. Instantiate a SelfDrivingLabDemo class.
  b. Perform optimizations for grid search, random search, and Bayesian optimization.

Methods video S7. Open the cloud-control Jupyter notebook via Google Colab and install the self-driving-lab-demo Python package, see steps 16 and 17

Methods video S8. Copy-paste the PICO ID from Thonny to Colab and control the setup remotely through the “evaluate” command, see steps 18 and 19

Methods video S9. Perform the “Hello, World!” of optimization, comparing grid search vs. random search vs. Bayesian optimization, see step 19

Methods video S10. Visualize the results of the optimization comparison, see step 19

## Expected outcomes

It is expected that users will successfully set up the hardware and software for a closed-loop experiment. Further, users will run their first “autonomous drive” given in an example interactive notebook and explore additional example notebooks.

**Figure 24:**
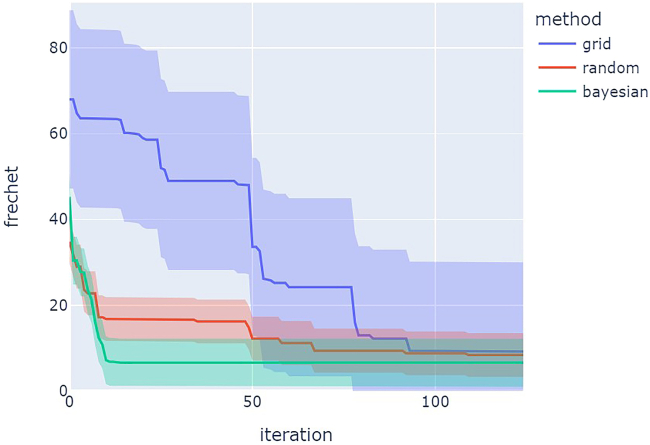
Example optimization comparison between grid search, random search, and Bayesian optimization averaged over repeated campaigns Error bands are the standard deviation across multiple trials. Lower Fréchet distance between observed and target spectra is better.

**Figure 25:**
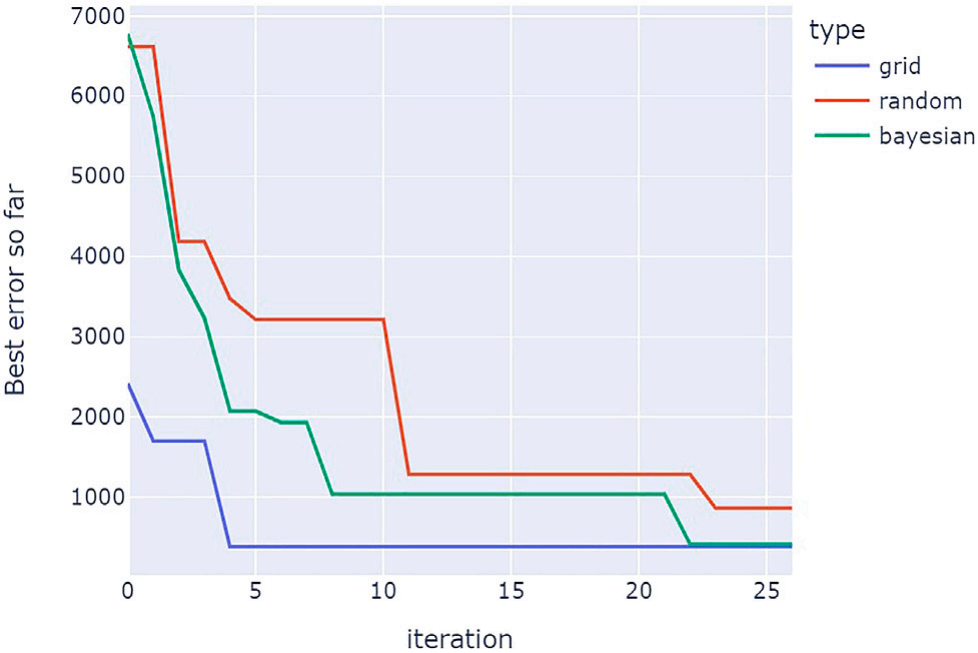
Example optimization comparison between grid search, random search, and Bayesian optimization Lower error is better.

**Figure 26:**
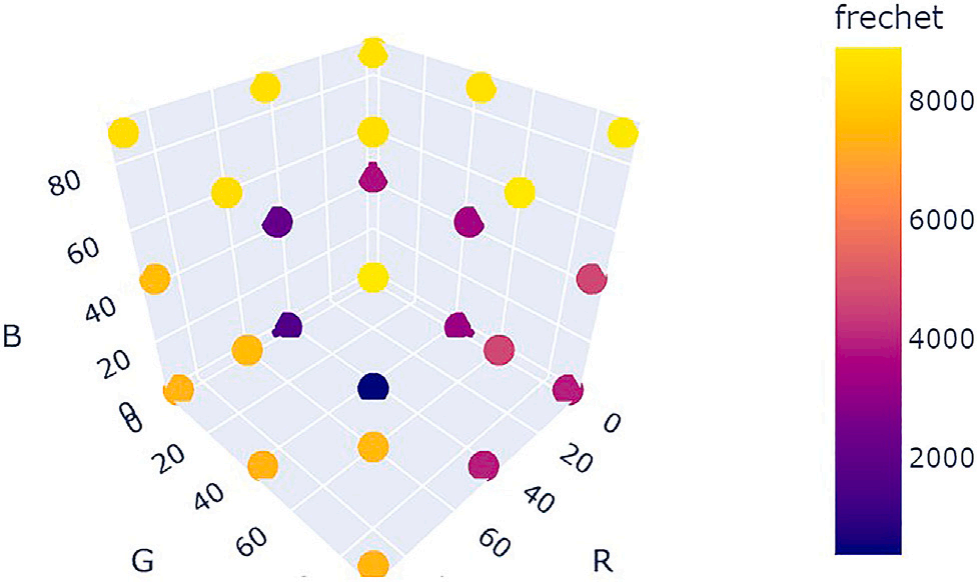
Twenty-seven grid search points colored by the Fréchet distance between the target spectrum and the sensor data evaluated at each grid point

**Figure 27:**
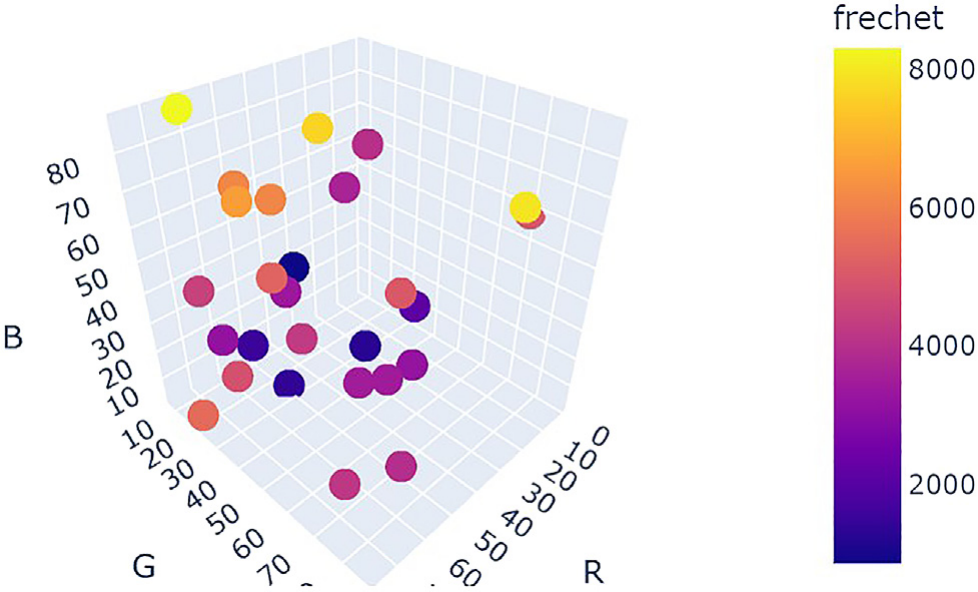
Twenty-seven random search points colored by the Fréchet distance between the target spectrum and the sensor data evaluated at each grid point

**Figure 28:**
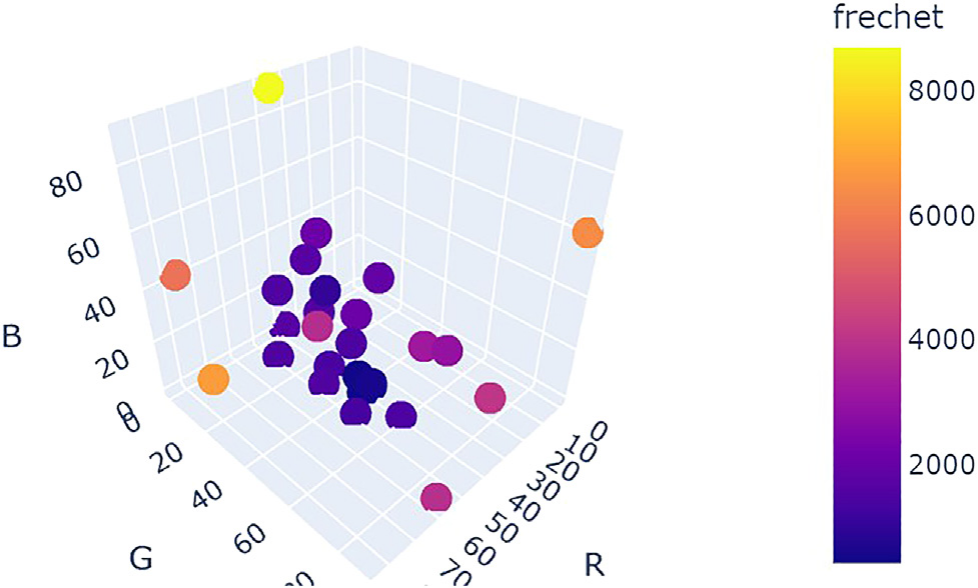
Twenty-seven Bayesian optimization points colored by the Fréchet distance between the target spectrum and the sensor data evaluated at each grid point

**Figure 29:**
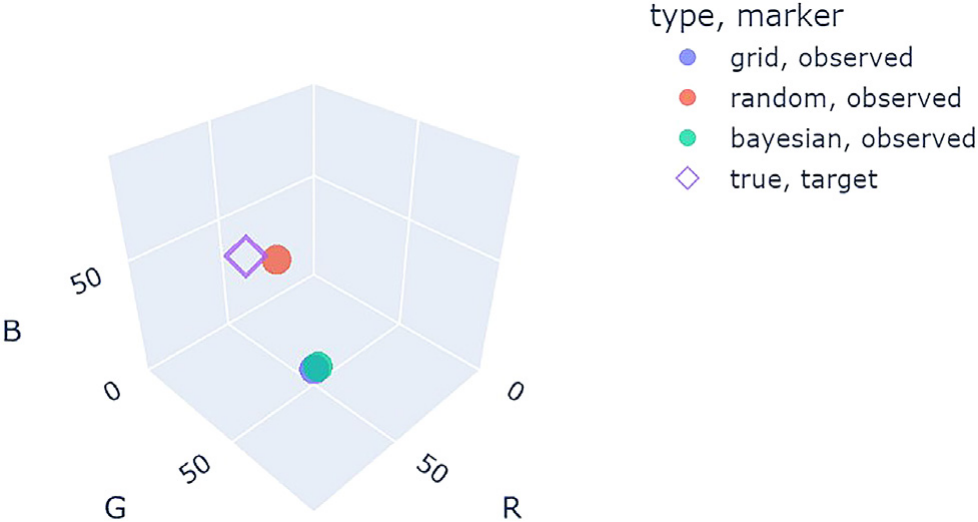
The true, underlying RGB target (purple diamond) and the best observed points for grid search (blue circle), random search (red circle), and Bayesian optimization (green circle) Bayesian optimization gave the closest match to the true target.

Figure 24 shows a comparison of optimization results for grid search vs. random search vs. Bayesian optimization averaged over repeat campaigns with standard deviation error bands, where Bayesian optimization, on average, performs the best. Figure 25 shows one of the outputs from the cloud-based control notebook of best error so far vs. iteration number comparing grid search vs. random search vs. Bayesian optimization. Typically, grid search is the least efficient, Bayesian optimization is the most efficient, and random search is somewhere in-between. Figures 26, 27, and 28 show the points that were searched for a given campaign for grid search, random search, and Bayesian optimization, respectively. Finally, Figure 29 shows the true, underlying target color (defined by red, green, and blue values) and the best parameter set based on minimizing error between the observed spectrum and the target spectrum for each of the optimization methods.

## Quantification and statistical analysis

Discrete Fréchet distance, as implemented in https://github.com/cjekel/similarity_measures, is used to assess the mismatch between the currently observed spectrum and the target spectrum, where the target spectrum is determined by arbitrarily choosing a random set of RGB values and measuring the sensor data for the fixed, random set of RGB values. Lower Fréchet distances correspond to better matches between the observed and target spectra (i.e., lower error).

An example JSON document logged to a MongoDB database backend containing experimental data for a single run is given as follows:

{

 "utc_timestamp": "2022-11-4 06:51:16",

 "ch510": 354,

 "ch620": 5671,

 "ch410": 188,

 "ch440": 3675,

 "ch583": 2756,

 "_input_message": {

  "_session_id": "542e6e80-9c50-4c41-95a5-832603b96238",

  "B": 31,

  "atime": 100,

  "gain": 128,

  "astep": 999,

  "_experiment_id": "9b50c819-db8f-476f-b601-dbe79e871a46",

  "G": 3,

  "integration_time": 280.78,

  "R": 41,

 },

 "onboard_temperature_K": 294.1085,

 "sd_card_ready": True,

 "ch470": 2827,

 "ch550": 498,

 "ch670": 277,

}

The experimental parameters for two JSON documents are given in Table 1.

**Table 1:** Example of data obtained from two experiments

utc_timestamp	onboard_temperature_K	R	G	B	atime	gain	astep	ch410	ch440	ch470	ch510	ch550	ch583	ch620	ch670
11/4/2022 6:40	292.7041	41	3	31	100	128	999	188	3674	2828	354	498	2748	5661	276
11/4/2022 6:51	294.1085	41	3	31	100	128	999	188	3675	2827	354	498	2756	5671	277

The LED parameters are red (R), green (G), blue (B). The sensor settings are atime, gain, astep (affects integration time and intensity). The measured output values are of the form “ch###” where the three digit number corresponds to the full-width half-max (FWHM) wavelength being measured.

The code for grid search, random search, and Bayesian optimization is hosted at https://github.com/sparks-baird/self-driving-lab-demo/blob/main/src/self_driving_lab_demo/utils/search.py [permalink].

## Limitations

Environmental noise (e.g., light conditions) and hardware variation (LED, sensor, sensor positioning, etc.) may affect the results obtained.

## Troubleshooting

See the GitHub issue tracker for existing known issues or to post a new issue. See the GitHub discussions for general questions and discussion.

### Problem 1

Can I use this with alternate microcontrollers or firmware?

### Potential solution

The hardware configuration and software were designed based on Raspberry Pi’s Pico Wireless (Pico W) microcontroller. Libraries exist for LED control and the AS7341 light sensor in CircuitPython and Arduino. The hardware and configuration and software can be adapted for other microcontrollers. Contributions at https://github.com/sparks-baird/self-driving-lab-demo/ are welcome. See order required parts.

### Problem 2

Can I use this without connecting to the internet?

### Potential solution

A simple example of wired communication between a computer and the microcontroller for the microcontroller host code and a Jupyter notebook tutorial (client) can be found at https://github.com/sparks-baird/self-driving-lab-demo/tree/main/src/extra/nonwireless [permalink] and https://github.com/sparks-baird/self-driving-lab-demo/blob/main/notebooks/5.0-nonwireless-search.ipynb [permalink], respectively. While possible with some modification, data communication via a USB cable is not actively supported for new releases of microcontroller host code nor the advanced tutorials. The status of this feature is being tracked at https://github.com/sparks-baird/self-driving-lab-demo/issues/193. For private, secure, wireless communication between the Pico W microcontroller and the client (e.g., Jupyter notebook running locally), a free, private HiveMQ instance can be set up per the instructions in Software Setup. For recommendations regarding connecting to a 2.4 GHz network (e.g., in university classroom settings) see https://github.com/sparks-baird/self-driving-lab-demo/discussions/83 and https://github.com/sparks-baird/self-driving-lab-demo/discussions/88. See also additional prerequisites.

### Problem 3

Can I use this without logging to a MongoDB backend?

### Potential solution

If the MongoDB credentials are left to their default dummy values in secrets.py, then logging to the MongoDB backend will fail and the device will simply notify the user rather than exit the program. In other words, the device will function normally without database logging. The same applies for logging to an onboard SD card. If an SD card is detected, the microcontroller will write backup data to it, otherwise this step will be skipped. See step 13.a.

### Problem 4

The Stemma-QT to Grove connectors (or other items) are out-of-stock.

### Potential solution

First, look at Adafruit and other vendors to see if it is available. Note that Cat#1528-4424-ND is incompatible with the Maker Pi Pico base due to the adapter housing blocking it from being plugged in fully. If no Stemma-QT to Grove connectors can be located, another alternative is using a Stemma-QT to header pin cable (DigiKey Cat#1528-4209-ND) and plugging directly into the GPIO pins that correspond to Grove Port #6 of the Maker Pi Pico base. For other items that may be out of stock on DigiKey or Adafruit, other vendors may be used (e.g., AS7341 light sensor from electromaker). See order required parts.

### Problem 5

The sculpting wire doesn’t fit through the mounting holes.

### Potential solution

Ensure that the outer diameter of the sculpting wire is 14 AWG or higher (i.e., 1.628 mm or thinner). Enameled wire (often advertised as sculpting wire) has a very thin coating, whereas electrical wiring typically has a non-negligible insulation thickness. Optionally, for a more modular setup, a single M2.5 binding post (Digikey Cat#36-8737-ND) can be used to clamp the wire via a single mounting hole instead of looping the wire through each of the mounting holes. See order required parts and step 3.

### Problem 6

My SD card isn’t being recognized.

### Potential solution

First, we note that use of the micro SD card is optional and serves the purpose of onboard backup data logging. The 128 MB micro SD card recommended in this work (DigiKey Cat#1528-5250-ND) has been tested with the rest of the components. First, try removing the micro SD card completely and reinsert it, making sure there is an audible “click”. If the microcontroller fails to detect the micro SD card, then there may be a defect in the micro SD card or the Maker Pi Pico base. Try ordering an extra micro SD card (same one recommended above), and if it suddenly works, you should be able to request a refund on the first SD card. If it still does not work, contact the seller of the Maker Pi Pico base to request a replacement. If not using the recommended SD card, the card formatting may be incompatible with MicroPython (see https://github.com/CytronTechnologies/MAKER-PI-PICO/issues/4). In this case, you will likely need to purchase a different type of SD card. See order required parts and step 3.

## Resource availability

### Lead contact

Further information and requests for resources and reagents should be directed to and will be fulfilled by the lead contact, Taylor D. Sparks sparks@eng.utah.edu.

### Materials availability

This study did not generate new unique reagents.

## Data Availability

The code generated during this study is available on GitHub: https://github.com/sparks-baird/self-driving-lab-demo. The recommended option for ordering parts is https://www.digikey.com/short/qztj2jt7 AND Pico W with pre-soldered headers to avoid soldering. Alternatively, https://www.digikey.com/short/vtzjbvr2 is a standalone DigiKey order, but requires soldering headers onto the Pico W. For parts that are unavailable, we encourage readers to search for alternative vendors such as Adafruit, CanaKit, or PiHut. For a full video build tutorial, please refer to https://youtu.be/D54yfxRSY6s. Code for grid search, random search, and Bayesian optimization is hosted at https://github.com/sparks-baird/self-driving-lab-demo/blob/main/src/self_driving_lab_demo/utils/search.py. A version of record for v0.8.2 is given in Zenodo: https://doi.org/10.5281/zenodo.7855493. To cite all versions, see Zenodo: https://doi.org/10.5281/zenodo.7855492. We encourage readers to use the latest version of the self-driving-lab-demo Python package and revert to v0.8.2 if breaking changes occur that prevent use of the latest package with the instructions in this protocol. We will make efforts to minimize changes that are backwards incompatible, and we would highly appreciate if users would check the existing issues (see both open and closed issues) and open a new issue in the GitHub issue tracker if not already present in the existing issues. A free GitHub account can be created to comment on existing issues or open new issues, and a GitHub markdown syntax guide is available. Alternatively, users may also contact the authors at the emails listed in the author affiliations.
